# Combinatorial therapeutic approaches of photodynamic therapy and immune checkpoint blockade for colon cancer treatment

**DOI:** 10.1186/s43556-022-00086-z

**Published:** 2022-08-17

**Authors:** Yang Hao, Chih Kit Chung, Zili Gu, Timo Schomann, Xiaoxu Dong, Ruben V. Huis in ‘t Veld, Marcel G. M. Camps, Peter ten Dijke, Ferry A. Ossendorp, Luis J. Cruz

**Affiliations:** 1 Translational Nanobiomaterials and Imaging (TNI) Group, Department of Radiology, Leiden University Medical Center, Albinusdreef 2, 2333 ZA Leiden, The Netherlands; 2 JeNaCell GmbH, Winzerlaer Straße 2, 07745 Jena, Germany; 3 Percuros B.V, Zernikedreef 8, 2333 CL Leiden, The Netherlands; 4 School of Chinese Material Medica, Beijing University of Chinese Medicine, Beijing, 102488 China; 5 Department of Ophthalmology, Leiden University Medical Centre (LUMC), Leiden, The Netherlands; 6 Department of Immunology, Leiden University Medical Center, Albinusdreef 2, 2333 ZA Leiden, The Netherlands; 7 Department of Cell and Chemical Biology and Oncode Institute, Leiden University Medical Center, Einthovenweg 20, 2300 RC Leiden, The Netherlands

**Keywords:** Immunogenic cell death, Immune checkpoint blockade inhibitors, Indocyanine green, Photodynamic therapy, Poloxamer 407 (P407) hydrogel

## Abstract

Photodynamic therapy (PDT) has shown impressive therapeutic effects on various types of cancers by reactive oxygen species (ROS) generation and induction of immune responses. However, under certain conditions, the immune responses induced by PDT are not always sufficient to eradicate the remaining tumor cells. On the other hand, the photosensitizer indocyanine green (ICG) can mediate PDT under near-infrared (NIR) illumination, thereby enhancing the penetration depth of the excitation light into the tumor. We found that ICG is rapidly taken up *in vitro* by colorectal MC38 and CT26 tumor cells and it promotes PDT-mediated cell-killing effects. Our results furthermore revealed that ICG induces immunogenic cell death (ICD), as dendritic cells (DCs) were found to engulf ICG-PDT-treated tumor cells and undergo phenotypic maturation. ICG accumulated in tumors 2 h after administration, as measured by fluorescence and photoacoustic imaging. Considering the advantages of ICG as a photosensitizer, we sought to design a therapy that combines PDT and immune checkpoint blockade to maximize tumor control. To this end, a 25% thermosensitive polymer 407 hydrogel was included as a co-delivery platform for this treatment scheme. NIR-PDT under 808 nm irradiation in combination with cytotoxic T-lymphocyte-associated protein 4 (CTLA4)/programmed death-ligand 1 (PD-L1) checkpoint blockade prolonged survival rate of colorectal tumor-bearing mice by inducing a series of immune responses, like the phagocytosis of tumor debris by macrophages and DCs, and induction of acute inflammation, leukocyte infiltration, maturation and activation of DCs. Altogether, our work presents a NIR-triggered PDT strategy in combination with immune checkpoint blockade. Compared to a single treatment, the combination treatment increased efficiency to inhibit solid tumor growth and improved the survival rate of tumor-bearing mice.

**Supplementary Information:**

The online version contains supplementary material available at https://doi.org/10.1186/s43556-022-00086-z.

## Introduction

As a cancer treatment modality, photodynamic therapy (PDT) has several advantages, such as being minimally invasive, inducing less side effects, and its spatio-temporal selectivity. After administering the photosensitizer, a light source is used to directly induce tumor cell death by generating toxic reactive oxygen species (ROS) and singlet oxygen (^1^O_2_) in combination with oxygen-provided energy [1]. Conventional light-triggered photosensitizers are mainly used clinically to treat superficial cancers because of the low tissue penetration of light and can only reach lesions with a depth of around 1 mm. In contrast, near-infrared (NIR) agent-supported PDT may overcome the low penetration efficiency of traditional visible light [2]. To this point, several studies report that indocyanine green (ICG), the only food and drug agency (FDA)-approved NIR tumor imaging agent, has an excellent safety profile and can be used as a potent photosensitizer for PDT [3].

PDT can indirectly induce vascular damage, depending on the type of photosensitizer and PDT dose, and elicit immune responses that mediate anti-tumor effects [4, 5]. It has been demonstrated that PDT-induced tumor cell death is immunogenic [6, 7]. Specifically, these dying tumor cells can not only act as tumor-associated (neo)antigens but also secrete more damage-associated molecular patterns (DAMPs), which can trigger systematic immune responses, involving dendritic cells (DCs), macrophages, and other immune cells [8, 9]. Several DAMPs have been shown to act as critical determinants in PDT-induced immune responses, for example by elevating calreticulin exposure (CRT) to the cancer cell surface [10–12], via heat shock proteins (HSP70 and HSP90) exposure on cancer cells [13–15], or extracellular adenosine triphosphate (ATP) [16, 17]. The immunity induced by PDT involves among others CD8^+^ T cells, which play a key role in eradicating the tumor cells. However, this immune response is not always sufficient to inhibit the growth of the remaining tumor cells that survived from PDT [18]. Moreover, the generated anti-tumor immunity might be counteracted by various immunosuppressive factors in the tumor microenvironment. For instance, infiltrating myeloid-derived suppressor cells (MDSCs) and regulatory T cells (Tregs) can inhibit the tumor killing efficiency of effector T cells, which can favor tumor recurrence [19, 20]. Therefore, our rational is that it may be beneficial to support PDT with other immunomodulatory agents, which becomes increasingly appreciated in patient-tailored anti-cancer therapies [21–23]. In this sense, immune checkpoint blockade therapy can be exploited to decrease the immunoregulatory suppression in the tumor microenvironment. This creates a potential for designing synergistic therapies that combine PDT with immune checkpoint inhibitory blockade strategies [18, 24].

However, one should be aware that immune checkpoint inhibitors (ICI) can give side effects when given freely. To meet these requirements, nanosystems might be well-suited. Nanoparticles not only protect therapeutic vectors and photosensitizers from degradation in the circulation and prolong their retention, but also reduce the required combination therapy dose, minimize the risk of severe systemic toxicity, and prolong the immune responses [25, 26]. Moreover, nanomedicine and cancer immunotherapy research has shown promising data with a variety of polymer-based nanoparticles and hydrogels. These studies have shown further improved anti-cancer effects using nanoparticles (NP)-based PDT in combination with post-ICI in combination therapy [27]. The use of nanoparticles to co-deliver ICG with therapeutic vectors (e.g., drugs, antigens, and adjuvants) could serve as *in situ* vaccines after irradiation, followed by ICI therapy, which is also able to inhibit tumor propagation, recurrence, and metastasis by enhancing the vaccine-induced systemic antitumor immune response [27]. Although, human serum albumin (HSA) NPs or Poly (lactic-co-glycolic acid) (PLGA) NPs co-delivery of photosensitizer with some forms of immune checkpoint therapies (like siRNA or protein) [28, 29]. The research of Poloxamer 407 (P407)-hydrogel in the field of checkpoint immunotherapy in combination with PDT is just beginning. This formulation was FDA approved for various pharmaceutical applications and is well suitable for ICI delivery with excellent biocompatibility and safety, factors that are crucial for safe immunotherapy [30]. But to date, only limited studies have deployed P407 hydrogels for combinatorial cancer immunotherapies (like ICI and PDT). Recently, we reported promising results of a P407-based thermosensitive hydrogel as a sustained delivery system for anti-CTLA4 immune checkpoint antibodies or doxorubicin as a monotherapy [25, 31]. P407 provides promising outlooks for further exploration of notably combinatorial therapies. Additionally, in previous studies, we observed that P407 hydrogel retained high ICG concentration at the injection site for as long as 7 days, compared to delivery in phosphate-buffered saline (PBS), which support the rationale to perform multiple-time PDT with a single photosensitizer injection to induce enhanced anti-tumor effects [31, 32]. Therefore, the possibility to co-deliver the most clinically used immune checkpoint inhibitory antibodies, such as Ipilimumab and Nivolumab with P407 thermosensitive hydrogels in combination with PS is novel and remains to be further explored.

The present study aims at comprehensively investigating the biological properties and PDT-mediated anti-cancer and immune capabilities of ICG in colorectal cancer cell lines and tumor models. To this end, we assessed the accumulation of ICG in tumor cells *in vitro* and *in vivo*, to get an impression of its clinical utility for PDT and diagnosis. We evaluated the optimal conditions for ICG-based PDT (ICG-PDT) in MC38 colon adenocarcinoma and CT26 colon cancer carcinoma cells and observed effective cancer cell killing effects of ICG-PDT. We also explored the immunological effects of PDT-induced cell death *in vitro*. In addition, we determined the tumor inhibitory potential of P407-based anti-CTLA-4 and/or anti-PD-L1 therapy combined with PDT in MC38 and CT26 tumor-bearing mice models. We found that this P407-supported combination can effectively inhibit tumor growth and prolong the survival time of tumor-bearing mice by activating anti-tumor immune responses after treatment. Our data show the promises of P407-based combinational treatment strategies of PDT and ICI for simultaneous diagnosis as well as for efficient tumor growth inhibition, whilst mitigating the adverse effects of checkpoint therapy.

## Results

### *ICG as a photosensitizer* in vitro

**Fig. 1:**
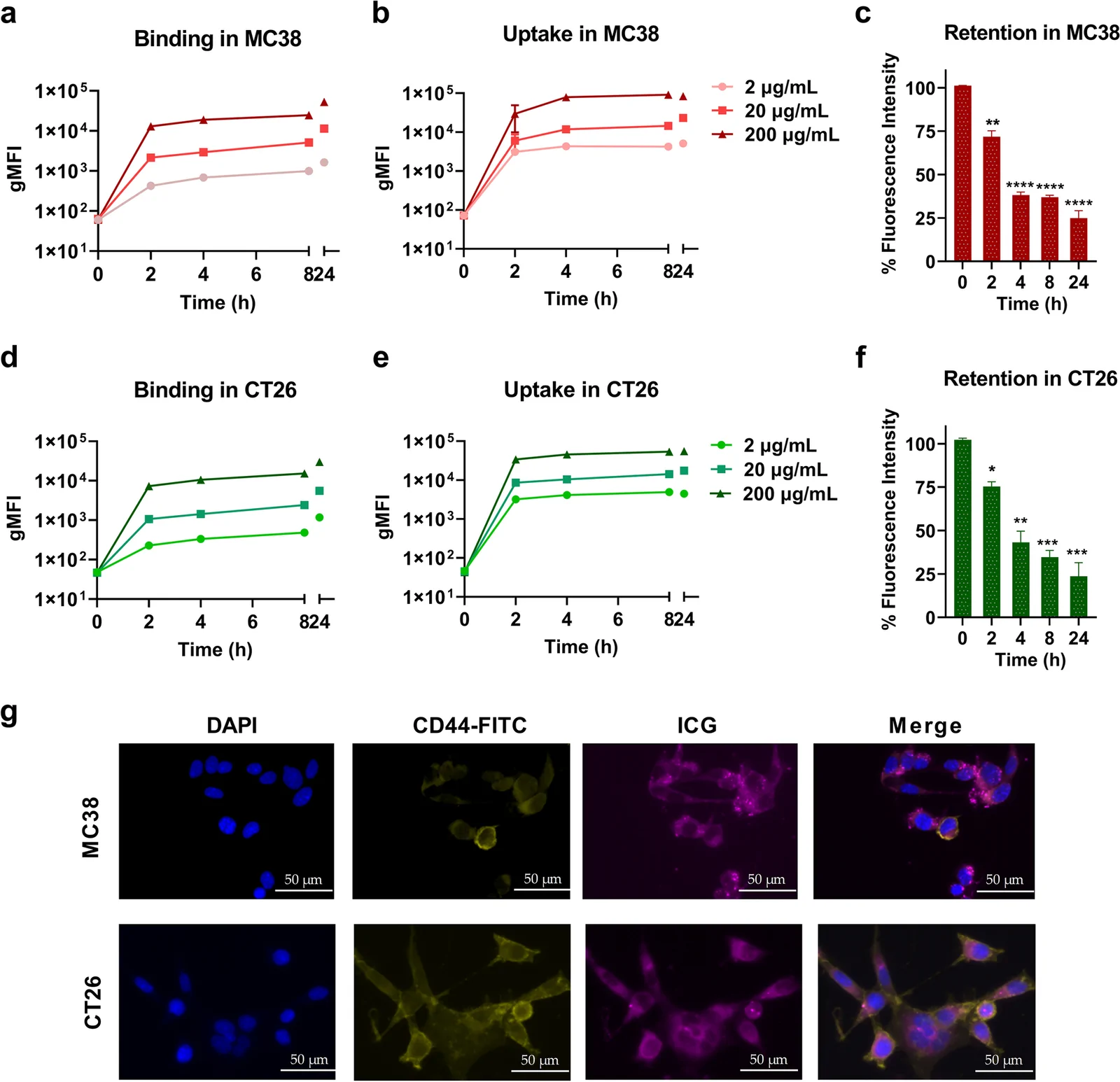
Cellular properties of the photosensitizer ICG *in vitro* (**a**) Cellular binding and (**b**) uptake assays with 2 µg/mL, 20 µg/mL, and 200 µg/mL ICG in MC38 cells over time by incubating cells with ICG at 4 °C and 37 °C, respectively. Detection was performed by flow cytometry and represented as gMFI of ICG. (**c**) Retention of ICG (50 µg/mL) in MC38 cells after 4 h co-incubation, collected cells were washed and detected by flow cytometry. (**d**) Cellular binding and (**e**) uptake assays with 2 µg/mL, 20 µg/mL, and 200 µg/mL ICG in CT26 cells over time by incubating cells with ICG at 37 °C and 4 °C, respectively. (**f**) Retention of ICG (50 µg/mL) in CT26 cells after 4 h co-incubation, collected cells were washed and detected by flow cytometry. The fluorescence signal positive population is shown as a percentage of CRC cells. (**g**) Fluorescence microscopy images of ICG (50 µg/mL)-treated MC38 and CT26 cells after 4 h of incubation. Scale bar = 50 μm

Traditional PDT showed beneficial anti-cancer effects on small colorectal tumors in a clinical quantitative pilot study [33]. The NIR agent ICG has an excitation wavelength of 808 nm which allows for a deeper penetration capacity in solid colorectal tumors and is therefore a promising photosensitizer for PDT. We first explored the uptake, binding, and retention kinetics of ICG in two colorectal cancer (CRC) cell lines to establish a working PDT protocol. To this end, MC38 and CT26 cells were incubated with different concentrations of ICG (2 µg/mL-200 µg/mL) and the geometric mean fluorescence intensity (gMFI) of cells was measured by flow cytometry. Temperature-dependent ICG binding to the cell membranes was investigated by incubating the cells at 4 °C. The binding studies showed a markedly lower fluorescence signal in MC38 (Fig. 1a) and CT26 cells (Fig. 1d), as compared to the signals from the uptake studies at 37 °C (Fig. 1b, e). The fluorescence in MC38 (Fig. 1b) and CT26 cells (Fig. 1e) increased in a concentration-dependent manner during 24 h co-incubating with ICG at 37 ℃. These results demonstrated that 4 h incubation would be sufficient for significant ICG accumulation in colon cancer cells *in vitro*. After a pulse of ICG exposure to cells, it remained in MC38 (Fig. 1c) and CT26 (Fig. 1f) cells, up to at least 8 h. To detect whether ICG was internalized into the cells, we performed immunofluorescence assays on CRCs 4 h post ICG treatment and found the intracellular presence of ICG in the cytoplasm for both cell lines (Fig. 1g). Taken together, our data show that ICG as a photosensitizer can be rapidly internalized by colorectal tumor cells *in vitro* and is retained in the cells for at least 24 h.

### *ICG supported photodynamic cytotoxicity of CRC cells* in vitro

**Fig. 2:**
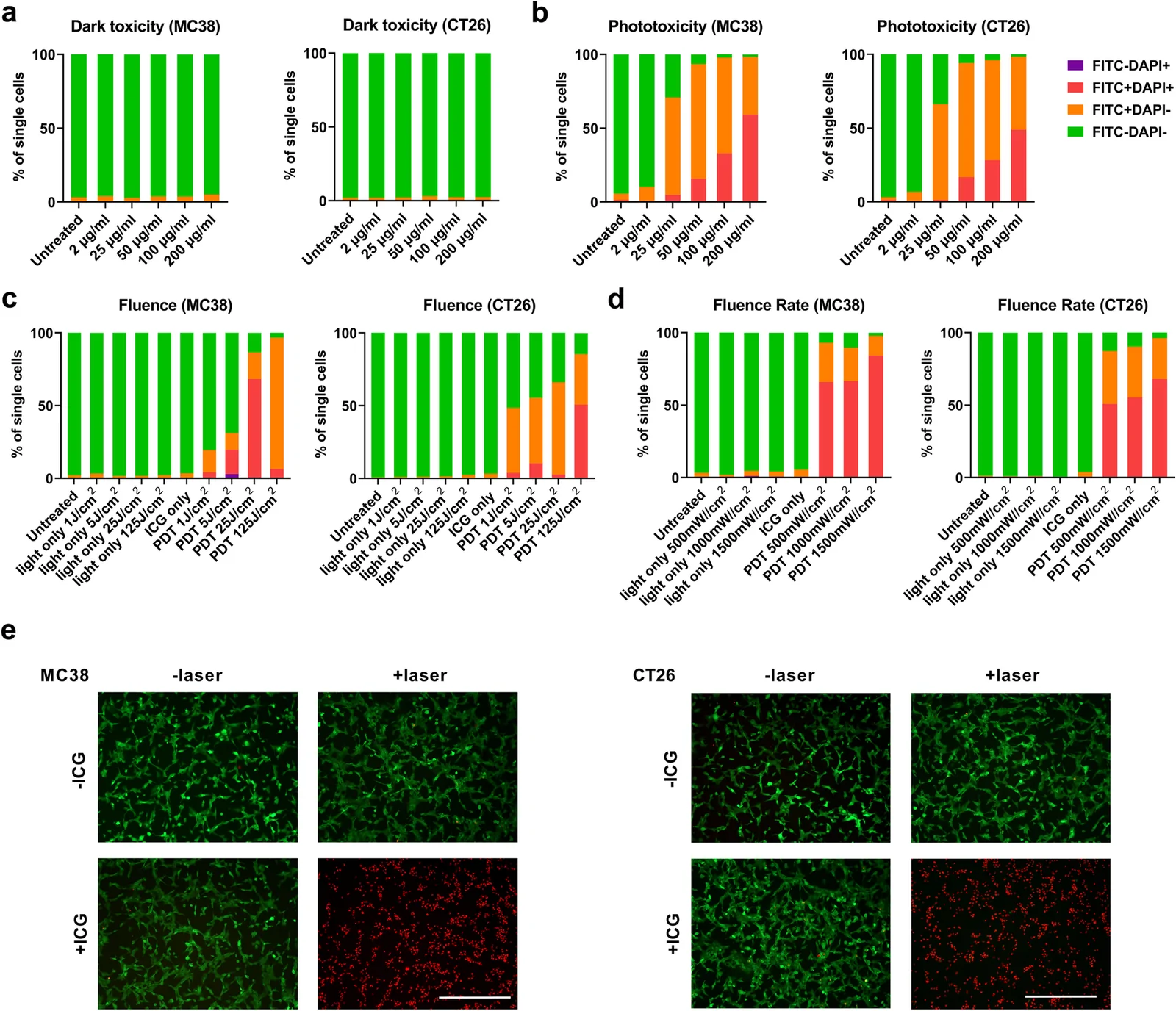
ICG-mediated cancer cell neutralizing effects *in vitro*. (**a**) MC38 cells and CT26 cells were incubated with ICG (2 µg/mL-200 µg/mL) for 4 h, followed by washing and overnight incubation to investigate the dark toxicity of ICG. The effect of (**b**) ICG concentration, (**c**) fluence, and (**d**) fluence rate in MC38 cells and CT26 cells, respectively, the *in vitro* cytotoxicity of ICG-PDT were determined by flow cytometry analysis after staining with DAPI and Annexin V-FITC. Single cells were gated into live cells (FITC^−^DAPI^−^), early apoptotic cells (FITC^+^DAPI^−^), late apoptotic cells (FITC^+^DAPI^+^) and necrotic cells (FITC^−^DAPI^+^). (**e**) PDT effects stained with LIVE/DEAD™ Viability/Cytotoxicity Kit. 10** × **magnification. Scale bar = 400 μm

First, we determined the toxicity of ICG on MC38 and CT26 cells with and without the absence of irradiation. The toxicity of ICG towards CRC cell lines was negligible at a concentration up to 200 µg/mL in MC38 cells and CT26 cells (Fig. 2a). When an irradiation with 808 nm laser at an intensity of 500 mW/cm^2^ for 125 J/cm^2^ was applied, ICG-based PDT (ICG-PDT) killed up to 90% MC38 or CT26 cells at concentrations of 50 µg/mL-200 µg/mL (Fig. 2b). PDT is known to generate ROS to kill tumor cells [1]. We found that ICG-PDT produced a notable increased ROS at ICG concentrations ranging from 20 µg/mL-200 µg/mL. This was accompanied by a significant level of dying tumor cells (Supplementary Fig. 1a, b) as validated cell apoptosis assays. In particular, the PDT dose required for killing CRC cells *in vitro* was investigated by 500 mW/cm^2^ for a total fluence of 1–125 J/cm^2^ and PDT for 125 J/cm^2^ at a fluence rate of 500–1500 mW/cm^2^ (illumination following 4 h incubation with 50 µg/mL ICG). As demonstrated in Fig. 2c, the laser itself could not induce cell death for a total fluence dose ranging from 1 J/cm^2^ to 125 J/cm^2^; however, after 4 h incubation with ICG, increased cell death up to 90% along with the fluence from 1 J/cm^2^ to 125 J/cm^2^, while the fluence for 125 J/cm^2^ induced complete cell death at fluence rate 500–1500 mW/cm^2^ (Fig. 2d). Based on these data, the living and dead cell viability assays were performed on CRC cells after PDT treatment. As shown in Fig. 2e, we observed that ICG-PDT treatment in both MC38 or CT26 cells induced sufficient cell death (red cells), while in the control group (PBS and light only), only a few damaged cells were visible. These data together show that ICG can act as a potential photosensitizer in CRC *in vitro* models with no obvious toxicity without laser irradiation at a concentration up to 200 µg/mL. PDT induced cell killing effects when cells were exposed to an 808 nm laser at a PDT dose of 125 J/cm2 and intensity ranging from 500 to 1500 mW/cm^2^.

#### ICG as an inducer of immunogenic cell death in CRC cells

**Fig. 3:**
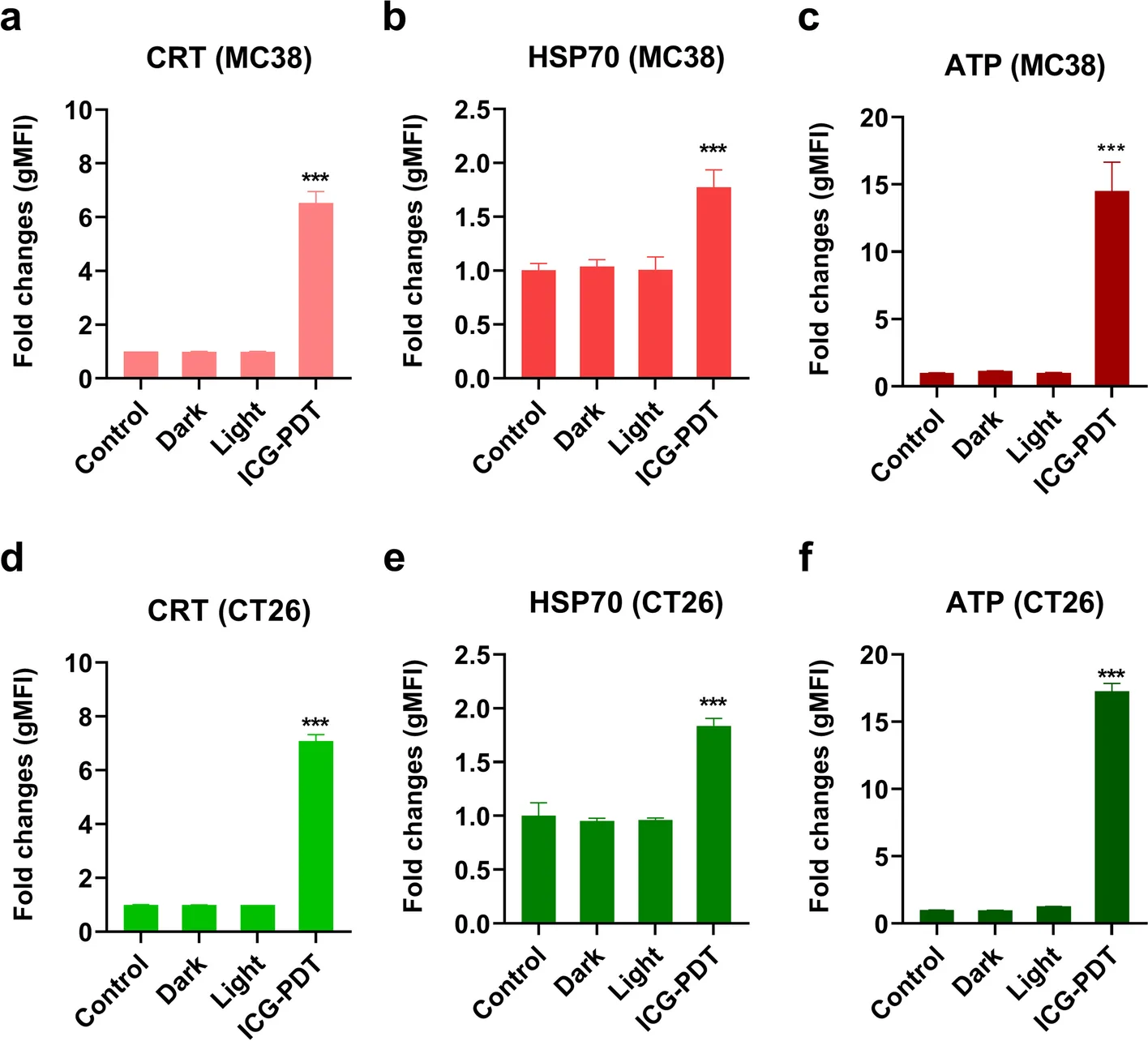
ICG-PDT-induced cell death in CRC cancer cells is associated with CRT and HSP70 exposure on the cell surface and ATP secretion. Fold change quantification of flow cytometry analysis of CRT exposure at the (**a**) MC38 cell surface and (**d**) CT26 cell surface of DAPI negative cells. Cells were harvested after 2 h of treatment with either ICG-PDT (irradiation by 808 nm laser was performed at 500 mW/cm^2^ for a total of 125 J/cm^2^ with 4 h incubation of 50 µg/mL ICG), ICG only (dark), irradiation only (light), 3 freeze/thaw (F/T) cycles at -20 °C or left untreated (control). Fold change quantification of flow cytometry analysis of HSP70 exposure at the (**b**) MC38 cell surface and (**e**) CT26 cell surface of DAPI negative cells. (**c**) MC38 cells and (**f**) CT26 cells were recovered 2 h after ICG-PDT, ICG only (dark), irradiation only (light), 3 freeze/thaw (F/T) cycles at -20 °C, or left untreated (control), and ATP was measured in the supernatants. ATP values represent a fold change relative to the untreated control group. All data shows the mean values ± SEM from three independent experiments. Statistical significance was calculated using the students *t*-test, by comparing experimental groups to the control and the differences are denoted as ns: not significant, *p < 0.05, ** *p* < 0.01, *** *p* < 0.001 and **** *p* < 0.0001

Studies have shown that a few photosensitizers can induce ICD, whose main characteristics are DAMPs (secreted or released molecules, and exposed molecules on their surface) signals from dying cells to enhance anti-tumor immune activation [34]. To this end, we explored the potential of ICG as an ICD inducer by analyzing the emission of decisive DAMPs, including surface CRT, surface HSP70, and extracellular ATP [34, 35]. MC38 and CT26 cells treated with PDT were stained with anti-CRT and DAPI 2 h after irradiation. We observed a significant six-fold and seven-fold increase in CRT exposure on the cell surface compared to untreated group in MC38 (Fig. 3a) and CT26 cells (Fig. 3d), respectively. Additionally, increased intracellular HSP70 exposure was found on the surface of MC38 (Fig. 3b) and CT26 cells (Fig. 3e), and higher ATP release in the extracellular environment (medium) (Fig. 3c, f). Our results demonstrated the possibility of ICG as the ICD inducer.

#### ICG-PDT treated cells induce phagocytosis, activation, and maturation of dendritic cells

**Fig. 4:**
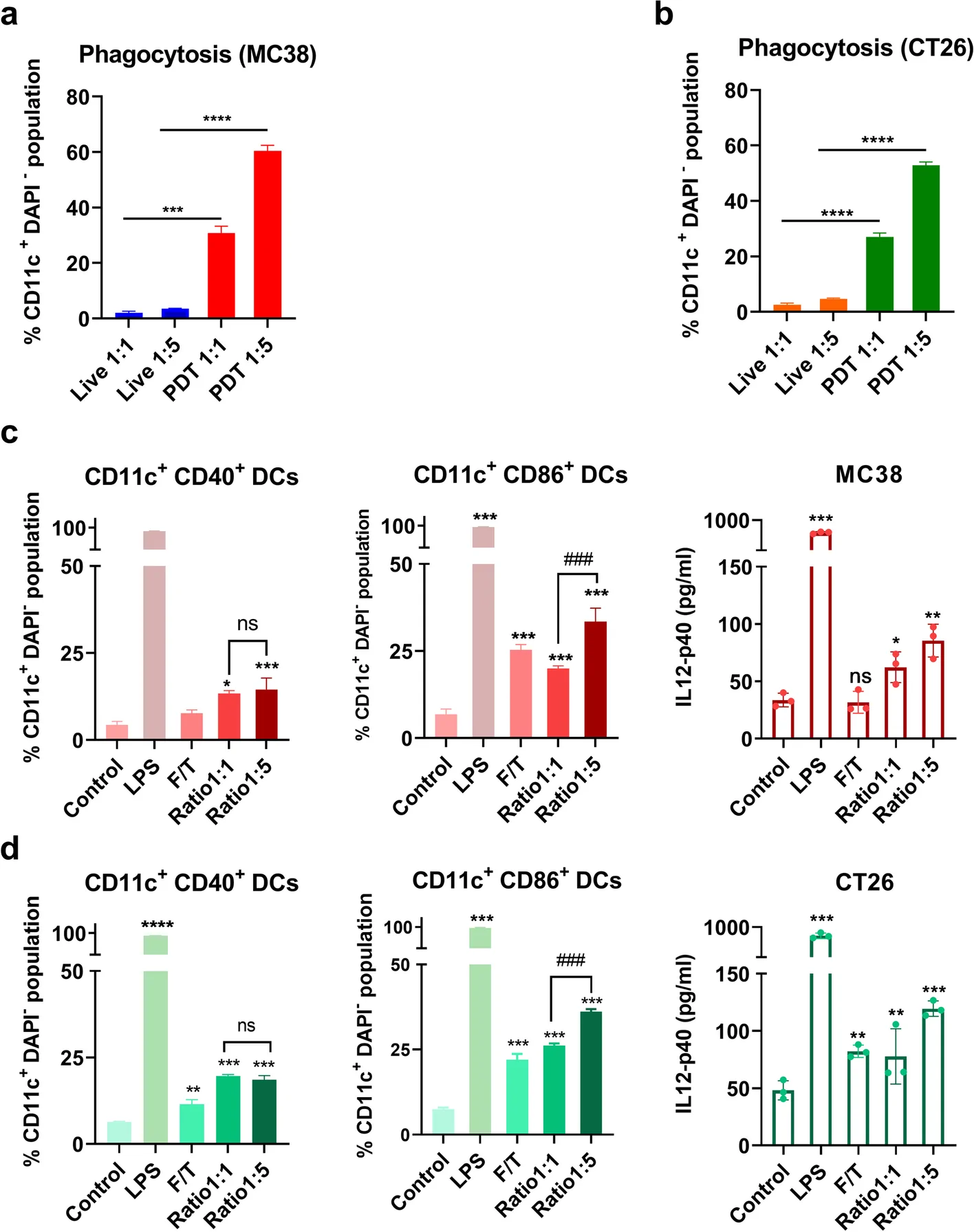
Immune-stimulating effects of ICG-PDT-induced cell death in colon cancer cells on DCs. Phagocytosis assay of (**a**) MC38 cells and (**b**) CT26 cells either after treatment with ICG-PDT or untreated were co-incubated with DCs *in vitro* immediately post-treatment for 2 h in two different ratios (1:1 and 1:5). The percentage of CD11c^+^CMFDA^+^ double-positive DCs that represent the phagocytosis DCs are shown as the mean values ± SEM. (**c**) The percentage of CD40^high^ and CD86^high^ cells in live DCs (CD11c^+^DAPI^−^ cells) and IL12-p40 expression in co-culture supernatant after 18 h co-culturing with treated or untreated MC38 tumor cells were compared to the untreated DCs control group. (**d**) The percentage of CD40^high^ and CD86^high^ cells in live DCs (CD11c^+^DAPI^−^ cells) and IL12-p40 expression in co-culture supernatant after 18 h co-culturing with treated or untreated CT26 tumor cells were compared to the untreated DCs control group. DCs incubation with 1 µg/mL LPS as a positive control or and untreated DCs as a negative control. All data showed the mean values ± SEM from three independent experiments. Statistical significance was calculated using a students *t*-test and the differences are denoted as ns: not significant, *p < 0.05, ** *p* < 0.01, *** *p* < 0.001 and **** *p* < 0.0001

Although we reported that ICG-PDT induces ICD *in vitro*, the effect of ICG-PDT treated tumor cells on immune cells in charge of stimulating an anti-tumor immunity remains not well understood and needs further exploration. Immature DCs have been reported to engulf PDT-induced tumor cell debris and activate DCs to a mature state, triggering the activation of T cells (undergo PDT-induced ICD) [36–39]. Thus, we analyzed the ability of DCs to phagocytose PDT-treated tumor cells. Tumor cells were stained with a fluorescent dye to monitor cell movement. We performed ICG-PDT treatment using the previously established *in vitro* PDT protocol in paragraph 4.2, after which ICG-PDT-treated cells were immediately added to DCs for 2 h co-culturing. We observed that ICG-PDT-treated MC38 cells were effectively engulfed by DCs compared to untreated live MC38 cells and the difference was significant between the co-culture cell ratio of 1:1 and 1:5 (Fig. 4a). The same phagocytic trend was seen in the co-culture assay with PDT-treated CT26 cells (Fig. 4b). Moreover, by measuring the co-stimulatory molecule CD40 and CD86 expression on the surface of the DCs and DC-secreted immune-related cytokines, interleukin (IL)-12-p40 in the co-culture medium, we study the indicative of the level of DCs maturation. DCs with E. coli lipopolysaccharide (LPS) was included as a positive control group and induced approximately complete activation of DCs (Fig. 4c, d). Similarly, ICG-PDT-treated cells induced DCs activation in a ratio-dependent manner, as evidenced by a higher percentage of mature DCs (CD11c^+^ CD40^+^ and CD11c^+^ CD86^+^) and the amount of IL-12-p40 in the medium (Fig. 4c, d) as was observed for the control cells and the cells subjected to three freeze/thaw cycles (F/T). Taken together, our results suggest that ICG as an ICD inducer can activate phenotypic maturation of DCs and their phagocytosis of post-PDT-treated tumor cells.

#### Optical imaging and photoacoustic imaging of ICG in tumor-bearing mice

**Fig. 5:**
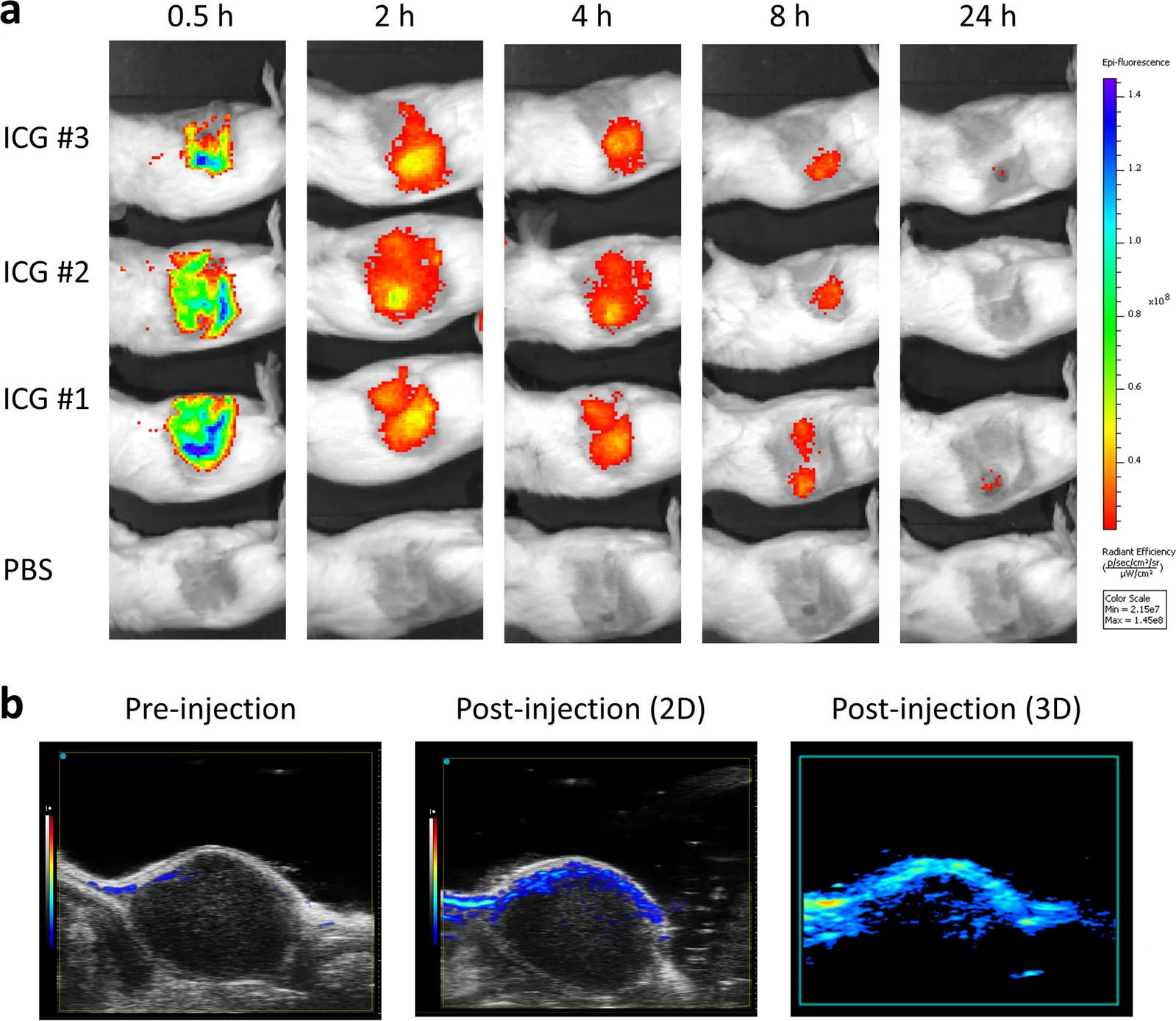
Fluorescence imaging and photoacoustic imaging of ICG *in vivo*. **a **Representative IVIS images of tumor-bearing mice 0.5 h, 2 h, 4 h, 8 h, and 24 h after administration of ICG (*n* = 3 per group). **b** Representative PA images of the tumor region were acquired pre-injection and 15 min post-injection

ICG shows absorption and fluorescence emission in the NIR wavelength region [40]. To evaluate the optical properties of ICG *in vivo*, CT26 tumor-bearing BALB/c mice were intravenously injected with ICG and analyzed by optical imaging. As shown in Fig. 5a, we found that ICG rapidly accumulated in tumors 2 h after injection and that the ICG fluorescence intensity diminished to the basal level after 24 h (Fig. 5a). ICG has been suggested as a photoacoustic (PA) imaging agent for its comparative fluorescence quantum yield [41]. Therefore, 15 min post-injection, PA spectra were acquired over a cross-section of the tumor (Fig. 5b). Taken together, the results demonstrated the ability of ICG application in CRC tumor monitoring by fluorescent imaging and PA imaging.

#### Physicochemical characterization and biological activity of ICG and immune checkpoint inhibitor (ICI) dual-loaded-P407 hydrogel

**Fig. 6:**
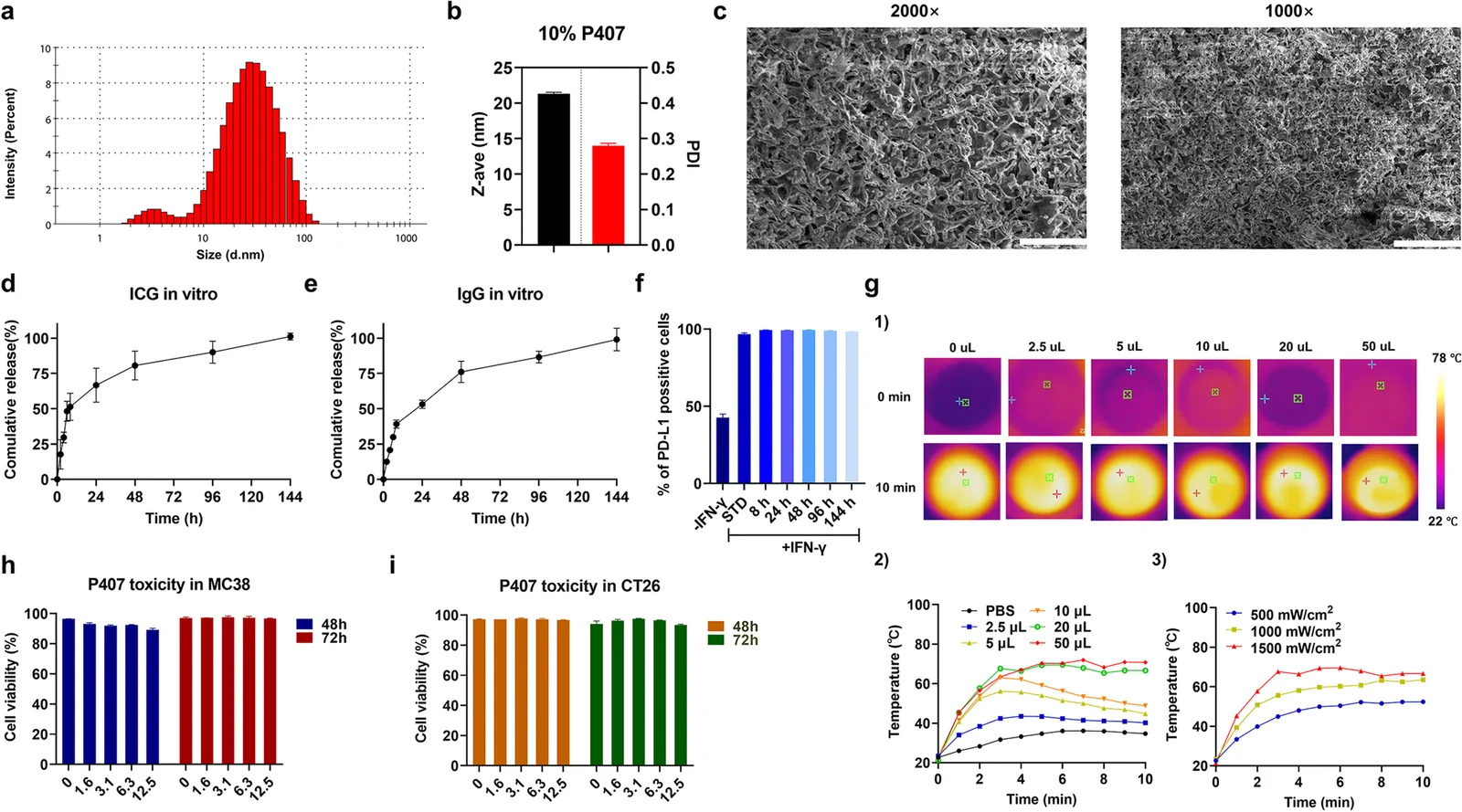
Physicochemical and biological characteristics of the antibodies and ICG dual-loaded P407 hydrogel *in vitro*. (**a**) Representative histogram for the micelle size of the P407 hydrogel. (**b**) The z-ave and PDI of the P407 hydrogel. (**c**) Typical SEM images of freeze-dried dual-loaded 25% P407 hydrogel, 2000 × (left panel, scale bar = 20 µm) and 1000 × (right panel, scale bar = 40 µm) magnifications. (**d**) *In vitro* human IgG release from 25% P407 hydrogel. (**e**) *In vitro* ICG release from 25% P407 hydrogel. (**f**) Immune checkpoint antibody binding efficiency after release from 25% P407 hydrogel. (**g**) Photothermal effects of ICG-loaded P407 hydrogel. (1) Typical thermal images of ICG-loaded P407 hydrogel; temperature changes of ICG-loaded P407 hydrogel at different time points with (2) various volume samples and (3) various fluence rates. (**h**) Viability of MC38 cells and (**i**) CT26 cells, analyzed by means of 7-Amino-Actinomycin D (7-AAD) analysis with flow cytometry 48 h or 72 h after incubation with P407 hydrogel. All data show the mean values ± SEM from three independent experiments

Based on our previous studies, we used 25% P407 hydrogel as a delivery system for a single anti-CTLA4 antibody or combined therapy for the current study [25, 31]. Here, we analyzed the physicochemical characteristics of the antibodies and ICG dual-loaded P407 hydrogel. The size and zeta potential measurement of our drug-loaded P407 nano-micelles were analyzed by dynamic light scattering (DLS). We found that dual-loaded hydrogel displayed a neutral surface (a zeta potential of 0.04 mV, not shown). In the DLS histogram, clearly, two distinctive peaks can be observed, the smaller peak at ~ 5 nm and the larger one at ~ 40 nm with an average size (z-ave) at ~ 20 nm (Fig. 6a,b). The smaller peak is related to the individual P407 micelles. However, at 10% P407 and room temperature micelles start to aggregate, which causes the larger peak. This size distribution clearly reflects P407 polymer and micelle chemical behavior after increasing the temperature and concentration. The sample measurement yielded a polydispersity index (PDI) of ~ 0.3 but this is mostly ascribed to the large size variation of the different micelle aggregates (Fig. b). Scanning electron microscope (SEM) analysis of lyophilized hydrogel structures showed that the hydrogel displayed highly connected structures with holes. The holes are a top view of the porous channels from which encapsulated agents can diffuse over time (Fig. 6c). *In vitro* release studies revealed that 25% p407 hydrogel displays sustained release kinetics of ICG and IgG, with around 75% IgG release being released after 48 h and the remainer being released within 144 h. (Fig. 6d). Similarly, around 67% of ICG released within 24 h, with the remaining ICG being released within 144 h (Fig. 6e). These data thus indicate that 25% P407 hydrogel could facilitate sustained release *in vitro*, up to 144 h. To confirm that the hydrogel does not impair the integrity and functionality of ICG and antibody, we investigated the binding efficiency of antibodies released from 25% P407 and the photothermal performance of ICG in the hydrogel. As shown in Fig. 6f, α-PD-L1 released from the hydrogel has a binding efficiency that is equal to the standard 5 µg/mL PD-L1 derived from the stock. For the experiment in Fig. 6g, the temperature was raised with increasing concentrations of the ICG-P407 suspension, and the temperature was increased to 74 °C in 10 min at a fluence rate of 1.5 W/cm^2^, which is as high as “free” ICG. Additionally, the P407 did not exhibit any direct cytotoxicity to MC38 (Fig. 6h) and CT26 (Fig. 6i) cells, even at the highest concentration tested. Taken together, these data show that P407 hydrogel is a promising platform for the delivery of ICG and checkpoint blocking antibodies.

#### ICG-PDT in combination with ICI improves survival of CRC tumor-bearing mice

**Fig. 7:**
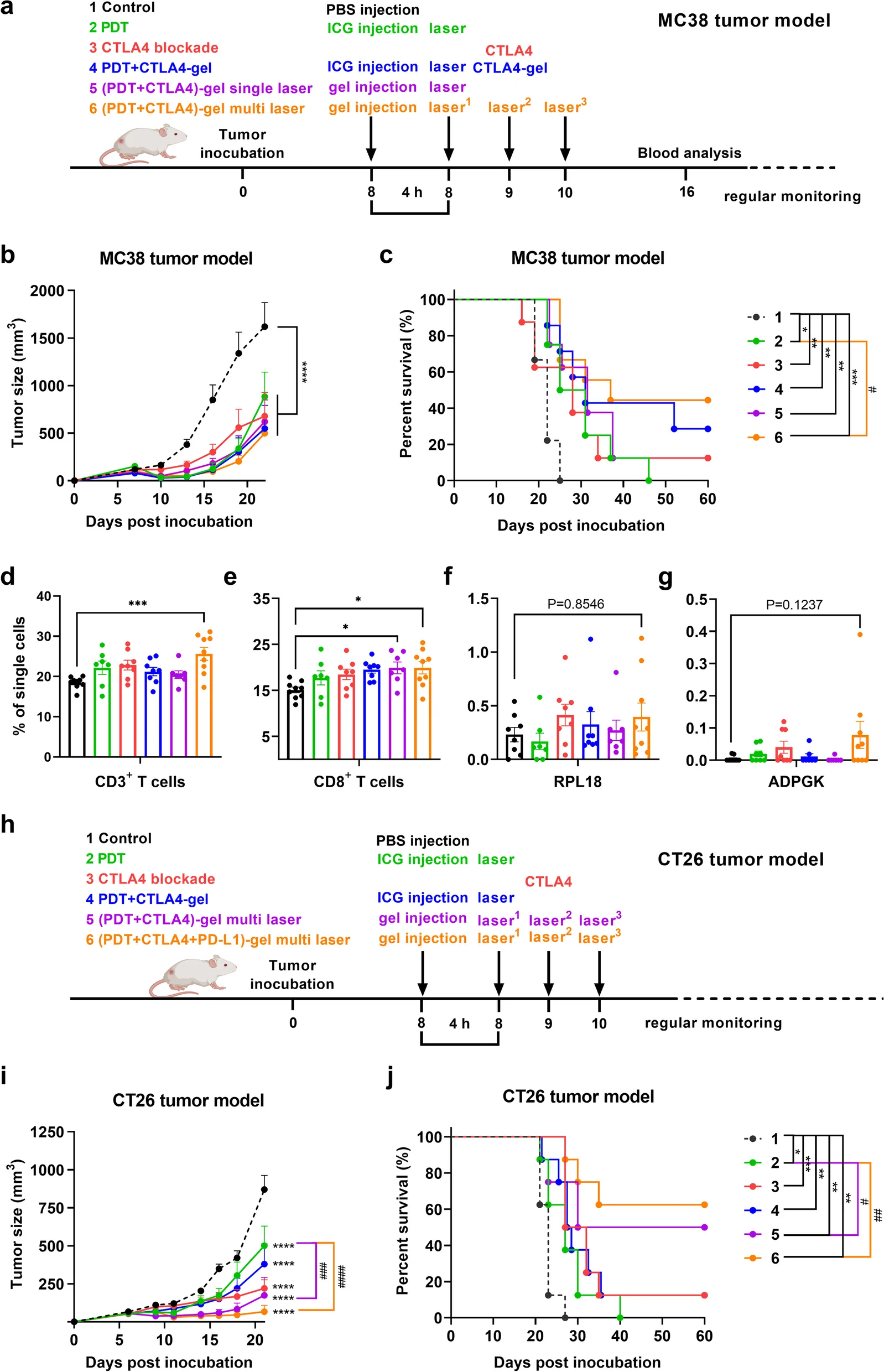
P407 hydrogel-based PDT in combination with CTLA4 blockade in colorectal tumor-bearing mice. (**a**) The experimental design of MC38 tumor-bearing mice model is depicted chronologically. (**b**) Average tumor size of MC38 tumor-bearing mice after treatment with group 1. PBS (Control), group 2. anti-CTLA4 blockade, group 3. ICG-supported PDT (PDT), group 4. PDT in combination with P407 hydrogel-supported CTLA4 blockade (PDT + CTLA4-gel), group 5. P407 hydrogel-supported PDT in combination with CTLA4 blockade with single laser treatment ((PDT + CTLA4)-gel) single laser), and group 6. P407 hydrogel-supported PDT in combination with CTLA4 blockade with multi-round laser treatments ((PDT + CTLA4)-gel)-multi laser) with the respective survival curves in (**c**). (**d**) The percentage of CD3^+^ T lymphocytes, (**e**) CD8^+^ T lymphocytes, (**f**) ADPGK-specific CD8^+^ T cells, and (**g**) RPL18-specific CD8^+^ T cells in the blood of mice eight days after treatment with either single therapies or combined therapy (n ≥ 7). (**h**) The experimental design of CT26 tumor-bearing mice model is depicted chronologically. (**i**) Average tumor size of CT26 tumor-bearing mice after treatment with group 1. PBS (Control), group 2. anti-CTLA4 blockade, group 3. ICG-supported PDT (PDT with single laser treatment), group 4. PDT in combination with P407 hydrogel-supported CTLA4 blockade (PDT with multi-round laser treatment + CTLA4-gel), group 5. P407 hydrogel-supported PDT combination with CTLA4 blockade ((PDT + CTLA4)-gel) with multi-round laser treatments), and group 6. P407 hydrogel-supported PDT in combination with dual-ICI blockade ((PDT + CTLA4 + PD-L1)-gel) with multi-round laser treatments) with associated survival curves in (**j**). Statistical significance for blood analysis was calculated using a one-way ANOVA, by comparing the experimental groups to the control; average tumor size analysis was performed with a two-way ANOVA; survival analysis was performed with a log-rank test (statistical differences are denoted as ns: not significant, *p < 0.05, ** *p* < 0.01, *** *p* < 0.001 and **** *p* < 0.0001)

The excellent tumor-killing effect of ICG-PDT *in vitro* provided us with inspiration to carry out a series of *in vivo* studies on mice with subcutaneous transplanted MC38 or CT26 cells to investigate the anti-tumor effect of ICG-PDT and the potential combination with CTLA4 antibody. Mice were inoculated with MC38 cells or CT26 cells and subjected to standalone treatments (ICG-PDT or anti-CTLA4 blockade) or P407 hydrogel-based combined therapy that was initiated on day 8, when the tumors were well established (Fig. 7a). As illustrated in Fig. 7b and Supplementary Fig. 2a, the tumor size in the control mice exhibited exponential growth in MC38 tumor-bearing mice, with tumors growing out 20 days post-inoculation. All treatments resulted in MC38 tumor growth delay. The therapeutic efficacy of the different treatments was furthermore analyzed with Kaplan–Meier survival curves (Fig. 7c and Supplementary Fig. 2a). We observed an improved survival rate in all P407 hydrogel-based combined therapy groups with negligible weight changes (Supplementary Fig. 2c) compared to the control group. The P407 hydrogel-based PDT combination therapy with CTLA4 blockade with multi-round treatment ((PDT + CTLA4)-gel)-multi laser) with a total fluence of 125 J/cm^2^ resulted in the strongest effects on survival compared to the PDT subgroup, which is in line with the previous reports for multi-round PDT [32].

We previously showed that PDT in combination with immunostimulators induced the exposure of neo-epitopes that can trigger systemic anti-tumor immune responses [42]. To test this hypothesis, T cell tetramer stainings were performed in the blood of MC38 tumor-bearing mice eight days post-PDT [43]. Whilst the percentage of both the CD3^+^ T cells (Fig. 7d) and CD8^+^ T cells (Fig. 7e) increased in all groups, this change was only significant for the (PDT + CTLA-4)-gel multi-laser treatment (both CD3^+^ and CD8^+^) and PDT + CTLA-4 gel treatment (only CD8^+^). The (PDT + CTLA-4)-gel multi laser treatment also induced more tumor neo-antigen (ADPGK and RPL18)-specific CD8^+^ T cells than the control group (Fig. 7f,g). However, this difference was not statistically significant. Next, the efficiency of hydrogel-mediated PDT combination therapy with immune checkpoint inhibitors was accessed in the CT26 colon cancer carcinoma tumor model (Fig. 7h), as in the MC38 model, a similar tumor growth delay was observed after all these aforementioned treatments (Supplementary Fig. 2b). Mice treated with P407 hydrogel-based multi-round PDT combination with anti-CTLA4 antibodies exhibited a comparable extent of tumor growth inhibition (Fig. 7i). This occurred without significant weight loss (Supplementary Fig. 2d), showing again that the treatment was well tolerated. The tumor inhibitory effects were found to be further improved by the combination of the anti-PD-L1 and anti-CTLA-4 antibodies (Fig. 7i), leading to significantly improved survival compared to mice without treatment and PDT single treatment (Fig. 7j*)*. Altogether, 25% P407 hydrogel-based PDT combination therapy with ICIs significantly increased the survival of CRC tumor-bearing mice.

#### ICG-PDT combination with ICI blockade modulates immune functions in lymphoid organs

**Fig. 8:**
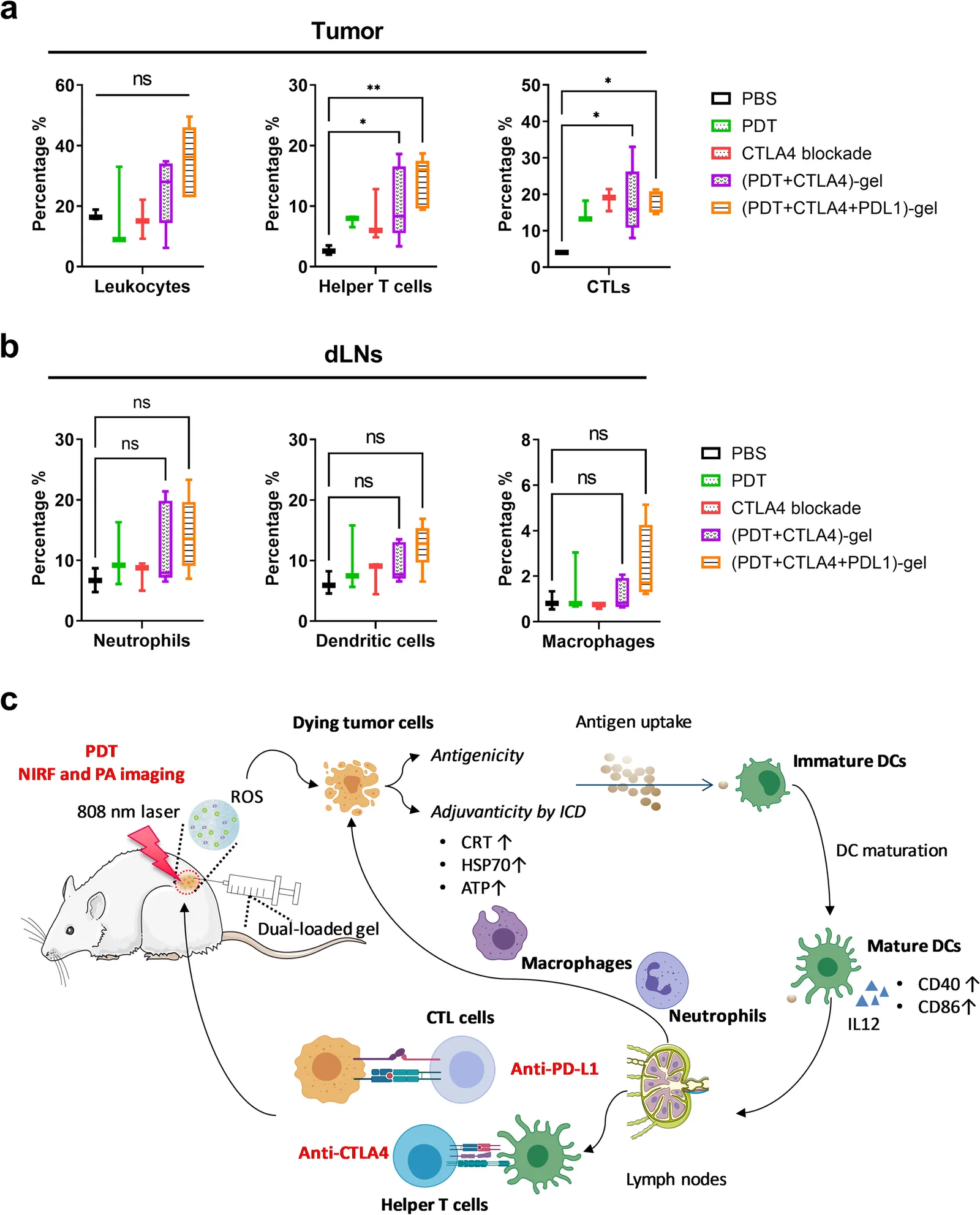
Analysis of immune cell populations in tumor and secondary lymphoid organs from treated MC38 tumor-bearing mice. The experimental design is depicted chronologically. Mice were inoculated with MC38 tumors (*n* = 5 mice per group) at day 0. Eight days post-inoculation, the tumors had been well established, and the mice were injected with different treatments as described in the methods section. Two days after the treatments, mice were sacrificed and organs were collected, processed, and stained for further analysis of the immune cell populations by means of flow cytometry. Gating was performed by FlowJo and included only live CD45.2^+^ cells. Populations were further gated to include helper T cells (CD3^+^ CD4^+^), CTLs (CD3^+^ CD8^+^), neutrophils (CD11b^+^Ly6G^+^), DCs (CD11b^+^CD11c^+^), and macrophages (CD11b^+^F4/80^+^). Cell populations in the tumor and dLNs are shown in (**a**) and (**b**), respectively. (Statistical differences are denoted as ns: not significant, *p < 0.05, ** *p* < 0.01, *** *p* < 0.001 and **** *p* < 0.0001). (**c**) Rational design of our current working network: P407 hydrogel-based synergistic PDT-immunotherapy for imaging and therapy

To investigate the possible alteration in the tumor microenvironment and secondary lymphoid organs upon the discussed treatments, we analyzed the diverse immune cell populations present in the tumor and lymph nodes of MC38 tumor-bearing mice. On day 11, organs were collected from mice received different treatments and the immune cell populations were analyzed (Fig. 8a). As shown in Fig. 8b, both single PDT and anti-CTLA4 antibodies treatment resulted in insignificant increase of helper T cells (CD4^+^ T cells) and cytotoxic T lymphocytes (CTLs) (CD8^+^ T cells) in tumor, whereas the combination treatment (including the (PDT + CTLA-4)-gel multi laser treatment and PDT + CTLA4 + PD-L1)-gel multi laser treatment) significantly increased the amount of CTLs in tumor microenvironment (Fig. 8b). The changes of helper T cells and CTLs and not significant in the tumor draining lymph nodes (dLNs) (Supplementary Fig. 3a, b). These results corroborate our previous study that CTLs are required for efficient treatment [18]. Compared to the mock-treated mice, PDT and its combination treatments induced rapid recruitment of neutrophils (CD11b^+^Ly6G^+^) to the dLNs (Fig. 8b). Previous studies showed that PDT prompts powerful acute inflammatory responses, including complement activation and accumulation of neutrophils and other inflammatory cells [8, 44, 45]. In the dLNs, we observed that the populations of DCs (CD11b^+^CD11c^+^) and macrophages (CD11b^+^F4/80^+^) were increased in groups treated with P407 hydrogel-based PDT in combination therapy with ICI accompanied by insignificant differences (Fig. 8b). In the spleen, the number of neutrophils, dendritic cells, and macrophages was found to be not different between the control and treated groups (Supplementary Fig. 3c, d,e). It has recently been shown that PDT combination therapy with immunomodulatory treatments can increase the number of mature inflammatory myeloid cells in MC38 tumor-bearing mice [42]. However, we did not observe significant changes in the levels of mature inflammatory monocytes (CD11b^+^F4/80^+^Ly6C^+^) and the immature myeloid population (CD11b^+^Ly6G^−^Ly6C^+^) in spleen, tumor, and dLNs after treatment with the combination (data not shown). As shown in Fig. 8d, a schematic overview explaining the cellular mechanisms underlying the combinatorial treatment, following local injection of a mix of ICG-CTLA4/PD-L1/P407 solution, the thermosensitive P407 formed an *in situ* hydrogel network in which ROS was generated by means of ICG irradiation with an 808 nm laser. This induced a series of immune responses.

## Discussion

PDT and immune checkpoint blockade can be effective to deal with certain cancer malignancies, however, as standalone treatment modalities, they bear numerous shortcomings. Furthermore, traditional checkpoint blockade treatment regimens were often associated with immune side effects, but recent nanotechnological insights might be considered to solve these issues. Therefore, in this study, we evaluated P407 hydrogel-based ICG-PDT combined with immune checkpoint blockade on two distinct murine colon cancer models. This co-delivery approach allows for, on the one hand, to reduce systemic antibody concentrations and, on the other hand, to simplify the route of agents’ administration to achieve multiple PDT and sustained antibody release. Our results demonstrated that this combination therapy facilitated efficient colorectal tumor growth inhibition and improved the survival rate and time of colorectal tumor-bearing mice compared to single treatments only.

We started with several *in vitro* ICG-PDT studies and characterized the hydrogel formulation. Our *in vitro* results are in line with previous studies and showed that ICG can be taken up by tumor cells in the cytoplasm (Fig. 1, Supplementary Fig. 4) [46]. Although ICG accumulated in tumor cells, we showed that, without proper light irradiation, ICG does not exhibit toxicity. However, when exposed to an 808 nm laser, ICG-PDT can induce complete cell death by generating ROS *in vitro *(Fig. 2, Supplementary Fig. 1), and this might lead to ROS-based stress of the endoplasmic reticulum and the activation of danger signaling pathways [47]. Our present study shows that ICG-PDT induces immunogenic cell death in MC38 or CT26 tumor cells. This immunogenicity was shown *in vitro *by DAMPs (secreted or released molecules, and exposed molecules on their surface) studies and DCs activation assays. Our results support the notion that ICG can induce CRT and HSP70 exposure on dying tumor cell surfaces as well as ATP release from tumor cells (Fig. 3). Subsequently, this triggers phagocytosis of tumor cells by DCs after PDT. In addition, dying tumor cells induced phenotypic maturation of DCs *in vitro* (Fig. 4). In MC38 tumor mice model, we further found that PDT treatment increased the expression levels of CD3^+^ T cells and CD8^+^ T cells in the circulating blood (Fig. 7d,e) and the infiltration of immune cells at the tumor site (Fig. 8a), although these changes were not significantly different. Taken together, our results are consistent with earlier studies with photosensitizers (hypericin [33], photosens and photodithazine [36], 5-ALA [48], Rose Bengal [49], glycoconjugated chlorin [50], pz I and pz III [37]) in which the data showed that PDT induces ICD, mainly through DAMPs signals from dying cells which could trigger anti-tumor immunity. Still more efforts are needed to explore the effect of ICG-PDT on ICD nature and associated DC activation *in vivo*.

Moreover, our results showed that ICG quickly accumulated at tumor sites as indicated by the strong NIR fluorescent signal 2 h post-intravenous injection (Fig. 5a) [51]. In addition, we obtained a strong PA imaging signal resulting from the ICG administration, which we did not observe before injection (Fig. 5b). This result is consistent with the previously published results obtained in subcutaneous xenograft mouse models of U-87MG and A431 human cancer cells [52]. Together, these data demonstrate that the various ICG imaging techniques can be leveraged as simple and efficient multimodal diagnostic and imaging tools, which can be applied before tumor treatment.

As we demonstrated the immunogenic effects of ICG-PDT, it makes sense to include immunomodulatory therapies as a support to facilitate synergistic tumor growth inhibition. Firstly, we explored the P407 hydrogel-based ICG-PDT combination with CTLA4 blockade therapy in the MC38 colon adenocarcinoma model. We observed that the ICG-PDT treatment alone had effects on tumor growth suppression when compared to untreated tumors, however the remaining tumor cells recurred one week after treatment (Fig. 7b, Supplementary Fig. 2). In comparison, ICG-PDT in combination with CTLA4 antibodies loaded P407 hydrogel injection in situ cocktail exhibited effective tumor suppression and moderate percentage of tumor ablation in MC38 tumor-bearing mice (Fig. 7b,c). While combinational P407 hydrogel-based ICG-PDT and CTLA-4 blockade therapy significantly reduced tumor growth when undergo multiple times irradiation, displayed the highest tumor elimination proportions associated with strong anti-tumor immune responses (Figs. 7, and 8). As reported recently by Wu Qinghua et al., the timing of ICI intervention after PDT plays an important role in the antitumor effect [53]. This P407-hydrogel-based combined therapy is based on repeated PDT and sustained anti-CTLA4 antibodies within 7 days which can be realized with a single dose of hydrogel, resulting less cumbersome treatment schemes, which potentially mitigates side effects and enhance antitumor efficiency by appropriate ICI administration time points after PDT. As shown in Supplementary Fig. 5, we demonstrated how P407 hydrogel significantly lower antibody levels in serum. Based on the promising results from the MC38-tumor tumor model, we continued to explore this treatment protocol in the CT26 murine colorectal tumor model. P407-hydrogel-based CTLA4 therapy resulted in equal tumor growth inhibition efficiency in both tumor models as the “free” CTLA4 blockade treatment, which is consistent with our previous *in vitro* data (Supplementary Fig. 6) and our previous statement [26]. All described single therapies or combined therapies had delayed CT26 tumor growth compared to the mock control group. The combination therapy (ICG-PDT-based combination with dual-ICI therapy (anti-CTLA4 and anti-PD-L1)) resulted in the strongest tumor growth inhibition and the highest percentage of tumor-free survival rate (62.5%). P407 hydrogel together with ICG-PDT and ICI demonstrated an equal or better efficacy than nanoparticles in combination with ICG-PDT and chemotherapeutic drugs (cisplatin [54], tirapazamine [55], Pt (IV) prodrugs [56], and doxorubicin [57]), ICG-PDT combined with chemotherapy and photothermal therapy, and ICG-PDT combined with anti-PD-L1 blockade [28].

Finally, we evaluated the effects of the combination treatment on immune cell populations in the tumor and lymph nodes in MC38 tumor-bearing mice. We have recently reported that dLNs play a pivotal role in PD-1/PD-L1 checkpoint blocking therapy in the MC38 and CT26 cancer models [58]. Here, our results showed that PDT only as well as the PDT-based combination treatment can induce rapid recruitment of inflammatory neutrophils, DCs, and macrophages in the dLNs (Fig. 8).This observation in accordance with other studies demonstrating that PDT can trigger acute inflammation [59], which might lead to both increased macrophage activation [44, 60, 61] and DCs maturation [28]. We observed that PDT in combination with ICI was associated with a significant increase number of CTLs and helper T cells in tumors, whereas PDT or CTLA-4 blockade alone did not (Fig. 8). We also found that the hydrogel-supported (ICG-PDT + anti-CTLA4 + anti-PD-L1) treatment had no obvious effects on the myeloid-derived suppressor cells in the tumor (data not shown), which is consistent with the observation of Liu et al*.* These authors reported that organic–inorganic NPs consisting of Ce6-PDT in combination with anti-PD-1 treatment significantly prolonged the survival of the mice, but not through repolarization of the tumor microenvironment [62]. Altogether, PDT and immunotherapy assisted with nanodelivery hydrogel systems can provide promising outlooks for cancer therapy development.

In summary, our data provide the rationale for the generation of ICG-PDT-based tumor vaccine or DC vaccine for cancer therapy. We reported a practical strategy that can facilitate more safe and efficient tumor growth inhibition by means of P407-mediated PDT in combination with CTLA4 and PD-L1 blockade. This approach takes advantage of the NIRF and PA imaging properties of ICG while ameliorating the tumor inhibitory effects and survival time in tumor-bearing mice. Based on our results, we hypothesize that our combination therapy induced infiltration of neutrophils and macrophages in the dLNs, leading to acute inflammation. We speculate on four distinct steps during the treatment that putatively underly the therapeutic effects of the combined therapy (represented schematically in Fig. 8c). In step 1), after local injection of ICG and ICI loaded with hydrogel, ICG-PDT first ablates the primary tumor by the generation of ROS. In step 2), the ICG-induced dying tumor cell debris can be regarded as tumor-derived (neo)antigens and phagocytosed by macrophages and dendritic cells. Step 3) ICG-PDT-induced immunogenic cell death and DAMPs release further induce acute inflammation and leukocyte infiltration, as well as maturation activation of DCs. Step 4) ICI help to enhance immune responses by decreasing immunoregulatory suppression. Overall, our results make important contributions to expand PDT-based combination therapy strategies, and the clinical application of biomaterials into cancer diagnosis and therapy strategies.

## Materials and methods

### Materials and reagents

Poloxamer 407 powder (catalog number: 9003–11-6) and ICG (catalog number: 3599–32-4) were purchased from Sigma Aldrich, USA. ICIs were obtained from BioXCell, USA. The catalog number of *InVivo*MAb anti-mouse CTLA4 (clone 9H10) and *InVivo*Plus anti-mouse PD-L1 (clone B7-H1) is BE0131 and BP0101, respectively. Murine MC38 (colon adenocarcinoma) and CT26 (colon cancer carcinoma) cells were cultured in IMDM medium (Lonza, USA) supplemented with 10% fetal calf serum (Sigma-Aldrich, USA), 2 mM l-glutamine (Gibco, UK), 25 mM β-mercaptoethanol (Sigma-Aldrich, USA), and 100 IU/mL penicillin/streptomycin (Gibco, UK) and cultured in an incubator (Panasonic, Japan) at 37 °C and 5% CO_2_. All cells used were mycoplasma and MAP-tested free. A 30% hydrogel was made by adding cold PBS (pH = 7.4) into P407 powder in a 50 mL Falcon tube and dissolved by stirring overnight at 4 °C. ICG was first dissolved in PBS, then ICG solution and antibodies were added into the clear 30% P407 hydrogel by mixing. Finally, an appropriate amount of PBS was added to make the final concentration of the P407 hydrogel 25%.

### Animals and tumor models

All animal experiments were designed according to the Code of Practice of the Dutch Animal Ethical Commission and subjected to the regulations as stated in project AVD116008045. The Animal Experimental Committee from the Leiden University Medical Center (LUMC) approved all experiments. Six to twelve weeks female C57BL/6 J mice or BALB/c mice were purchased from Harlan Laboratories (ENVIGO, the Netherlands) and housed in pathogen-free animal facilities in LUMC.

### Uptake, binding, and retention of ICG *in vitro*

MC38 or CT26 cells were seeded in 96-well plates (Greiner, the Nederland) at 7 to 10 × 10^3^ cells per well and were allowed to attach overnight. Tumor cells were co-incubated with ICG (2 µg/mL, 20 µg/mL, and 200 µg/mL) for a specified time at 37 °C and 4 °C, respectively, for the uptake assay and the binding assay. For the binding experiments, cells were pre-cooled at 4 °C for 4 h and then co-incubated with ICG for a specified time at 4 °C. Collected cells were washed in PBS 3 times before fixing in 1% formalin (J.T. Baker) at 4 °C for 15 min. After fixation, washed cells were reconstituted in fluorescence-activated PBA buffer (PBS supplied with 0.5% bovine serum albumin (BSA) and 0.02% sodium azide) for LSRII flow cytometer analysis (BD Biosciences, USA). Cells were then collected by the same procedure as described for the uptake experiments using flow cytometer analysis. For the retention experiment, cells were co-incubated with 20 µg/mL ICG for 4 h and were subsequently washed three times in PBS and thereafter refreshed with photosensitizer (ICG) free medium. After a specific time (0 h, 2 h, 4 h, 8 h, and 24 h), cells were collected following the same procedure as for the uptake experiments and analyzed by an LSRII flow cytometer.

### Intracellular fluorescence imaging of ICG in tumor cells

For the intracellular fluorescence imaging, MC38/CT26 cells were seeded on a micro cover glass-based 24-well plate at 4 to 5 × 10^4^ cells per well, and cells were allowed to attach overnight at 37͘ °C. Cells were thereafter incubated with 20 µg/mL ICG for 4 h, and subsequently washed in PBS and stained with 50 μg/mL CD44-FITC (Invitrogen, USA) antibody at 4 °C for 60 min. CD44 is typically expressed in cell membranes in multiple types of malignant tumor cells [63], which was used here to mark the tumor cell membrane. Next, cells were washed and stained with 0.25 μM 4′,6-diamidino-2-phenylindole (DAPI) (Sigma-Aldrich, USA) for 5 min at room temperature (RT). After staining with DAPI, cells on micro cover glasses were carefully washed five times in PBS to make the background clear and then mounted with Mowiol mounting medium (Sigma-Aldrich, USA) supplemented with 2.5% w/v DABCO (Merck). The microcover glasses were finally sealed with nail polish on the ground edges glass slides. The specimens were imaged on a Leica DM5500 B fluorescence microscope.

### PDT effects of ICG in tumor cells

MC38/CT26 (4 to 5 × 10^4^ cells per well) cells were seeded in a 24-well plate and thereafter co-incubated with increasing concentrations of ICG (2 µg/mL-200 µg/mL) for 4 h, washed three times with PBS to remove ICG that was not taken up, and thereafter supplied with fresh medium. Then, to access ICG mediated undesired toxicity, cells were culture without any other treatments; To study ICG mediated phototoxicity, tumor cells were irradiated by an 808 nm laser (Changchun New Industries Optoelectronics Technology, China) at a fluence rate of 500 mW/cm^2^ for a total fluence of 125 J/cm^2^ was applied after incubations with different indicated concentrations of ICG. At 24 h post-PDT, the apoptosis resulting from the PDT was determined by staining cells with Annexin V-FITC (Invitrogen, USA) and DAPI in Annexin V binding buffer for 20 min on ice before analysis by an LSRII flow cytometer. The data were analyzed using FlowJo™ v10.7.1 (BD Biosciences, USA). To explore the effects of fluence rate of irradiation on PDT toxicity efficiency, PDT was performed by an 808 nm laser at a fluence rate of 500, 1000, 1500 mW/cm^2^ for a total fluence of 125 J/cm^2^ after 4 h incubations with 50 ug/mL ICG. At 24 h post-PDT, cells were collected and analyzed similarly as previous described. To explore the effects of fluence dose of irradiation on PDT toxicity efficiency, PDT was performed by an 808 nm laser at a fluence rate of 500 mW/cm^2^ for a total fluence of 1, 5, 25, and 125 J/cm^2^ after 4 h incubations with 50 µg/mL ICG. At 24 h post-PDT, cells were collected and analyzed similarly as previous described. The imaging of live and dead cells after *in vitro* PDT using ICG was carried out by staining cells with Ethidium homodimer-1 and Calcein AM based on the operating protocol of LIVE/DEAD Viability/Cytotoxicity Kit (ThermoFisher, USA), and later imaged on a Leica DM5500 B fluorescence microscope.

### Immunogenic cell death (ICD) analysis of tumor cells after ICG-PDT

PDT was performed as described above: irradiation of tumor cells was performed using an 808 nm laser at a fluence rate of 500 mW/cm^2^ for a total fluence of 125 J/cm^2^ after 4 h of incubation with 50 µg/mL ICG. Supernatants of cultured cells were collected 2 h post-PDT for ATP release measurement according to manufacturer’s protocol (CellTiter-Glo® Luminescent Cell Viability Assay kit; Promega, USA) and the luminescence was measured on SpectraMax ID3 microplate reader (Molecular Devices, USA). Tumor cells were collected to analyze CRT and HSP70 exposure at the cell surface. The CRT exposure was analyzed with recombinant anti-CRT-Alexa Fluor® 488 antibody (Clone EPR3924, Abcam, UK), and the HSP70 exposure was analyzed with anti-HSP70-Alexa Fluor® 488 (Clone W27, Biolegend, USA) on an LSR-II cytometer.

### Phagocytosis assay

MC38 and CT26 colorectal cells were labelled with 1 μM CellTracker Green 5-Chloromethylfluorescein diacetate (CMFDA) (Abcam, UK) in serum-free media for 30 min. Thereafter, CMFDA-labelled MC38/CT26 cells were seeded into a 6-well plate and allowed to attach overnight. The next day, tumor cells were treated with ICG-PDT, as described above using an 808 nm laser at a fluence rate of 500 mW/cm^2^ for a total fluence of 125 J/cm^2^ after 4 h of incubation with 50 of ICG ug/mL. Thereafter, cells were collected, washed, and co-cultured with D1DCs (DC cells D1 [64]) in ratios of 1:1 or 1:5 for 2 h. After co-culturing, collected cells were washed in PBS, stained with anti-CD11c-BV605 (clone HL3, eBioscience, USA), and then reconstituted in FACS buffer before analysis by an LSRII flow cytometer. Engulfment of CMFDA-positive dead tumor cell debris by DCs was determined by quantifying the CD11c^+^ CMFDA^+^ cell population.

### Dendritic cell activation and maturation study

In brief, 5 × 10^4^ DCs were seeded in a 96-well plate and co-incubated with dying tumor cells stimulated with ICG-PDT (irradiation by 808 nm laser was performed at 500 mW/cm^2^ for a total of 125 J/cm^2^ with 4 h incubation of 50 µg/mL ICG) in ratios 1:1 or 1:5 for 18 h. 1 µg/mL LPS and F/T tumor cells (tumor cells challenged by multiple freeze–thaw cycles) were set as parallel controls. After co-culture, the supernatants were collected for IL-12 measurement with a standard sandwich enzyme-linked immunosorbent assay (ELISA); the cells were collected for analysis with anti-CD40-APC (Clone3/23, Biolegend, USA), anti-CD86-FITC (clone GL1, eBioscience, USA), anti-CD11c-BV605. DAPI was used to exclude dead cells on an LSR-II cytometer, and data was analyzed using FlowJo™ v10.7.1.

### *In vivo* fluorescence imaging and photoacoustic imaging

1 × 10^5^ CT26 tumor cells in 100 μL PBS were inoculated subcutaneously into the right flanks of 6–12-week-old female mice. After 10 days, a total of 8 mg/kg ICG was administered to tumor-bearing mice whose skin was shaved surrounding the tumor area. At the indicated time points (0.5 h, 2 h, 4 h, 8 h and 24 h), fluorescence spectrometry imaging was performed using the IVIS Spectrum (PerkinElmer, USA) under isoflurane anesthesia and analyzed with the Living Image IVIS software. Vevo LAZR-X (FUJIFILM VisualSonics, Japan) was used to obtain the PA and B-mode ultrasound images. For this measurement, an MX550D transducer was used with a center transmit of 40 MHz, and an axial resolution of 40 μm. Photoacoustic spectra were gained over a cross-section of the tumor at 796 nm, and the data was analyzed by Vevo LAB 5.5.0.

### ROS generation of ICG-PDT in tumor cells

MC38/CT26 (7 to 10 × 10^3^ per well) cells were seeded in a 96-well black plate (Thermo Fisher, Catalog#165,305) and challenged with indocyanine green (ICG) (2 µg/mL-200 µg/mL) for 4 h. Cells were washed several times to remove ICG. Thereafter, a fresh medium was added, and cells were irradiated with an 808 nm laser at a fluence rate of 500 mW/cm^2^ for a total fluence of 125 J/cm^2^. After PDT cells were collected in a 96-well v-bottom plate and stained with 10 µM 2',7'-Dichlorodihydrofluorescein diacetate (DCFH-DA) (Abcam, Catalog#ab113851) for 30 min. Thereafter, cells were washed for 3 time with PBS and transferred to a 96-well black plate before analyzed with the SpectraMax ID3 microplate reader with excitation at 485 nm and emission at 535 nm.

### Characteristics of dual-loaded P407 hydrogel

Dual-loaded P407 hydrogel particles were subjected to DLS, which was measured using Malvern Zetasizer (Nano ZS, Malvern Ltd., UK). This allows the analysis of the z-ave and PDI of dual-loaded P407 hydrogel particles. For this purpose, 10% P407 solutions were prepared by adding cold PBS (pH = 7.4) into P407 powder in a 50 mL Falcon tube and dissolved by stirring overnight at 4 °C. Scanning electron microscopy (SEM) images of lyophilized hydrogel formulations were obtained using a NovaSEM 450 to determine the morphology of hydrogel particles.

### Toxicity of P407 hydrogel

The toxicity of P407 hydrogel to tumor cells was measured using an LSR-II cytometer (BD Biosciences). After co-culturing with different concentrations of P407 granules, cells were stained with 7-AAD (Invitrogen, Waltham, USA-#A1310) before flow cytometry analysis.

### Binding efficiency of released samples

The measurement for *in vitro* release of ICG and human IgG from 25% P407 hydrogel was performed as described before [25]. To assess the antibody binding efficiency of released immune checkpoint antibodies from the hydrogel, released samples from P407 hydrogel loaded with mouse anti-PD-L1 antibody (10F.9G2 Biolegend®, 5 µg/mL) were obtained at indicated time points by placing the hydrogels at 4 °C, taking aliquots at different time points and refreshing with the same amount of PBS. Attached B16F10 mouse melanoma cells were stimulated with interferon (IFN)-γ (20 IU/µL; Biolegend®) for 24 h to upregulate their PD-L1 expression. Thereafter, collected B16F10 cells were first stained with either purified PD-L1 antibodies released from hydrogel (5 µg/mL) or “free” anti-PD-L1 (5 µg/mL) that was included as a positive control, then stained with the secondary antibody (Donkey-anti-Rat IgG 488) before flow cytometry analysis. PD-L1 binding levels were calculated as a measure of the mean fluorescence intensity of the secondary antibody. Flow cytometry measurements were performed on LSR-II Flow-cytometer (BD Biosciences), and the data were analyzed using FlowJo LLC version 10 software.

### The photothermal heating efficiency of released samples

To measure the *in vitro* photothermal heating efficiency, aqueous solutions of different volumes (0, 2.5 µL, 5 µL, 10 µL, 20 µL, and 50 µL) from hydrogel solutions (ICG concentration in the hydrogel is 2.67 mg/mL) were introduced into PBS to a total volume of 0.5 mL in a 24-well cell culture plate. Thereafter, the samples in the plate were irradiated with 808 nm NIR laser at different power densities (0.5, 1.0, 1.5 W/cm^2^) for 10 min, respectively. The temperatures of each of the solutions were measured every 1 min with a digital thermometer with a Hikvision temperature hand scanner (DS-2TPH10-3AUF, China). PBS was chosen as a control.

### PDT and P407-based combination therapeutic treatments *in vivo*

4 × 10^5^ MC38 cells or 1 × 10^5^ CT26 tumor cells in 100 μL PBS were injected into the right flanks of C57BL/6 J mice and BALB/c mice, respectively. On day eight, mice with established MC38 tumors (average size approximately 100 mm^3^) were randomly divided into seven subgroups (n ≥ 7): control (PBS), anti-CTLA4 blockade, ICG-supported PDT (PDT), PDT in combination with P407 hydrogel-supported CTLA4 blockade (PDT + CTLA4-gel), P407 hydrogel-supported PDT in combination with CTLA4 blockade with a single round treatment ((PDT + CTLA4)-gel) single laser), and P407 hydrogel-supported PDT in combination with CTLA4 blockade with a multi-round treatment ((PDT + CTLA4)-gel)-multi laser). The dose of ICG and CTLA4 antibodies was 8 mg/kg and 50 µg/mice (1.25 mg/kg), respectively. Before PDT, the fur surrounding the tumor area was shaved to minimize light absorption. PDT was performed under isoflurane anesthesia at a fluence rate of 500 mW/cm^2^ for a fluence of 125 J/cm^2^ at 808 nm at 4 h post-administration in PDT group, PDT + CTLA-gel, and (PDT + CTLA4)-gel)-single laser group or 3 times at 4 h, 24 h, and 72 h post-administration, represented as (PDT + CTLA4)-gel)-multi laser. In the CT26 mice model, the PDT was performed in a similar way as described in the MC38 model by 500 mW/cm^2^ for a fluence of 125 J/cm^2^ at 808 nm at 4 h post-administration in the PDT group and PDT + CTLA-gel group, while multi-round PDT at 4 h, 24 h, and 72 h post-administration for a fluence of 125 J/cm^2^ in the (PDT + CTLA4)-gel) group and the (PDT + CTLA4 + PD-L1)-gel) group for a total fluence of 125 J/cm^2^. From this point onwards, tumor size (length × width × height; expressed as mm^3^), body weight, and other general welfare aspects were monitored regularly until the end of the experiments.

### Blood analysis for tumor-specific immune responses

Blood analysis was performed by collecting 50 μL caudal vein blood of MC38 tumor-bearing mice on day 16. A total of 160 μL lysis buffer was added to the collected blood in Microvette® CB 300 blood collection tubes (Sarstedt, Germany) to remove red blood cells. Cells were then stained with Tetramer mix (PE-labelled ADPGK tetramer and APC-labelled RPL18 tetramer) on ice for 20 min. Thereafter, the cells were stained with anti-CD8α-BV421 (clone 53–6.7, Biolegend, USA), anti-CD3-PE-cy7 (clone 145-2C11, Thermo Fisher, USA). Finally, the cells were analyzed on an LSR-II cytometer, and the data were analyzed using FlowJo LLC version 10 software.

### Tumor, spleen, and lymph node analysis for immune cell populations

Based on our established protocol for immune cell population analysis in the tumor, spleen, and lymph nodes [65], the mice were sacrificed, and organs were resected and analyzed *ex vivo* on day 11. Tumors were cut into small pieces before *ex vivo* analysis. Then liberase TL (Roche, Germany) in the FCS-free medium was added to the sliced tumor pieces at 37 °C for 15 min. Single-cell suspensions of tumors, spleens and lymph nodes were acquired following the same procedure: by gently grinding the organs through a 70 μm cell strainer (Falcon, USA). Single-cell suspensions were equally divided into two parts. One was stained with lymphoid marker panel antibodies, including DAPI to exclude dead cells and antibodies against immune cell surface markers, including anti-CD45.2-APC eFluor 780 (clone 104, eBioscience, USA), anti-CD3-FITC (145-2C11, BD Bioscience, USA), anti-CD4-Brilliant Violet 605 (clone RM4-5, Biologend, USA), and anti-CD8α-APC-R700 (clone 53–6.7, BD Bioscience, USA). The other part of the single-cell suspensions was stained with DAPI to exclude dead cells and antibodies against myeloid immune cell surface markers, including anti-CD45.2-APC eFluor 780 (clone 104, eBioscience, USA); anti-CD11b-eFluor 450 (clone M1/70, eBioscience, USA); anti-F4/80-FITC (clone BM8, Biolegend, USA); anti-Ly6G-APC (clone RB6-8C5, BD Biosciences, USA); anti-Ly6C-Brillian Violet 605 (clone HK1.4, Biolegend, USA), and anti-CD11c-PE (clone HL3, BD Biosciences, USA). Cells were then analyzed on an Aurora 5-laser flow cytometer (Cytek, USA) and the data was analyzed with FlowJo LLC version 10 software.

### Statistical analysis

All data and graphs were generated and analyzed using GraphPad Prism software version 9.0 (La Jolla, USA). Statistical differences are analyzed by unpaired two-tailed student's *t*-test or one-way ANOVA unless otherwise stated and are denoted as ns: not significant, *p < 0.05, ** *p* < 0.01, *** *p* < 0.001 and **** *p* < 0.0001.

## Supplementary Information

**Additional file 1: Supplementary Fig. 1.** ROS generated by ICG-mediated PDT. **a** MC38 cells and **b** CT26 colon cancer cells were incubated with ICG (2 µg/mL-200 µg/mL) for 4 h, followed by PDT. Thereafter, the cells were harvested, washed, and stained with DCFH-DA to determine ROS generation of ICG-PDT. The data show the mean values ± SEM from one representative out of three independent experiments. Statistical significance was calculated using the students *t*-test, by comparing the experimental groups to the control (ns: not significant, *p*> 0.05, * *p* < 0.05, ** *p* < 0.01, *** *p* < 0.001 and **** *p* < 0.0001). **Supplementary Fig. 2.** Effects of P407 hydrogel-based PDT when combined with ICIs *in vivo*. **a** Individual tumor growth curve of MC38 tumor-bearing mice with associated weight changes of described groups in **c**; **b** Individual tumor growth curve of CT26 tumor-bearing mice with associated weight changes of described groups in **d**. (ns: not significant, *p* > 0.05, * or ^#^ *p* < 0.05, ** *p* < 0.01, *** *p* < 0.001 and **** *p* < 0.0001). **Supplementary Fig. 3.** Analysis of immune cell populations in dLNs and spleen from treated mice. Gating was performed in FlowJo and included only CD45.2^+^ cells. Populations were further gated to include helper T cells (CD3^+^ CD4^+^), CTLs (CD3^+^ CD8^+^), neutrophils (CD11b^+^Ly6G^+^), dendritic cells (CD11b^+^CD11c^+^), and macrophages (CD11b^+^F4/80^+^). **a** Helper T cell population in the dLNs; **b** CTL population in the dLNs; **c** neutrophils, **d** dendritic cells, and **e** macrophage population in the spleen. Statistical significance was calculated using a one-way ANOVA, by comparing the experimental groups to the control (ns: not significant, *p* > 0.05, * or ^#^ *p*< 0.05, ** *p* < 0.01, *** *p* < 0.001 and **** *p* < 0.0001). **Supplementary Fig. 4. **ICG was rapidly absorbed in tumor cells and gets distributed in different cytoplasmic organelles. After cells were incubated with ICG for 4 h, live cells were stained by different organelle markers, then fixed and photographed using fluorescence microscopy. ICG (yellow), Organelles (red) and nuclei (blue), Mito, mitochondria; Lyso, lysosomes; Golgi, Golgi apparatus; ER, endoplasmic reticulum. Scale bar = 50 μm. **Supplementary Fig. 5. **Serum CTLA4 antibody levels at days 0, 1, 3, and 6 following the administration of 50 μg CTLA4-loaded 25% P407 hydrogel. **Supplementary Fig. 6. **Analysis of the therapeutic effect of 25% P407 hydrogel-based CTLA4 therapy. **a** Individual tumor growth curves of MC38-tumor-bearing mice during the observed period with associated survival curves in **c. b **Individual tumor growth curves of CT26-tumor-bearing mice during the observed period with associated survival curves in **d**.

## Data Availability

All data needed to evaluate the conclusions in the paper are present in the paper and/or the Supplementary Materials.

## Abbreviations

PDT: Photodynamic therapy
ICG: Indocyanine green
NIR: Near-infrared
ICD: Immunogenic cell death
DCs: Dendritic cells
CTLA4: Cytotoxic T-lymphocyte-associated protein 4
PD-L1: Programmed death-ligand 1
P407: Poloxamer 407
ROS: Reactive oxygen species
^1^O_2_: Singlet oxygen
DAMPs: Damage-associated molecular patterns
CRT: Calreticulin
HSP: Heat shock proteins
ATP: Adenosine triphosphate
MDSCs: Myeloid-derived suppressor cells
Tregs: Regulatory T cells
ICI: Immune checkpoint inhibitors
NP: Nanoparticles
HAS: Human serum albumin
PLGA: Poly (lactic-co-glycolic acid)
PBS: Phosphate-buffered saline
CRC: Colorectal cancer
Gmfi: Geometric mean fluorescence intensity
IL: Interleukin
PA: Photoacoustic
DLS: Dynamic light scattering
z-ave: Average size
PDI: Polydispersity index
SEM: Scanning electron microscope
CTLs: Cytotoxic T lymphocytes
dLNs: Tumor draining lymph nodes
7-AAD: 7-Amino-Actinomycin D
