# Supplementary material for: Combinatorial therapeutic approaches of photodynamic therapy and immune checkpoint blockade for colon cancer treatment
Source: Mol Biomed. 2022 Aug 17;3:26. doi: 10.1186/s43556-022-00086-z (PMC9381671; doi:10.1186/s43556-022-00086-z)
Supplement: Supplementary file 1 — Additional file 1: Supplementary Fig. 1. ROS generated by ICG-mediated PDT. a MC38 cells and b CT26 colon cancer cells were incubated with ICG (2 µg/mL-200 µg/mL) for 4 h, followed by PDT. Thereafter, the cells were harvested, washed, and stained with DCFH-DA to determine ROS generation of ICG-PDT. The data show the mean values ± SEM from one representative out of three independent experiments. Statistical significance was calculated using the students t-test, by comparing the experimental groups to the control (ns: not significant, p> 0.05, * p < 0.05, ** p < 0.01, *** p < 0.001 and **** p < 0.0001). Supplementary Fig. 2. Effects of P407 hydrogel-based PDT when combined with ICIs in vivo. a Individual tumor growth curve of MC38 tumor-bearing mice with associated weight changes of described groups in c; b Individual tumor growth curve of CT26 tumor-bearing mice with associated weight changes of described groups in d. (ns: not significant, p > 0.05, * or # p < 0.05, ** p < 0.01, *** p < 0.001 and **** p < 0.0001). Supplementary Fig. 3. Analysis of immune cell populations in dLNs and spleen from treated mice. Gating was performed in FlowJo and included only CD45.2+ cells. Populations were further gated to include helper T cells (CD3+ CD4+), CTLs (CD3+ CD8+), neutrophils (CD11b+Ly6G+), dendritic cells (CD11b+CD11c+), and macrophages (CD11b+F4/80+). a Helper T cell population in the dLNs; b CTL population in the dLNs; c neutrophils, d dendritic cells, and e macrophage population in the spleen. Statistical significance was calculated using a one-way ANOVA, by comparing the experimental groups to the control (ns: not significant, p > 0.05, * or # p< 0.05, ** p < 0.01, *** p < 0.001 and **** p < 0.0001). Supplementary Fig. 4. ICG was rapidly absorbed in tumor cells and gets distributed in different cytoplasmic organelles. After cells were incubated with ICG for 4 h, live cells were stained by different organelle markers, then fixed and photographed using fluorescence micro [file 43556_2022_86_MOESM1_ESM.docx]

Supporting Information

Combinatorial Therapeutic Approaches of Photodynamic Therapy and Immune Checkpoint Blockade for Cancer Treatment

Yang Hao^1^, Chih Kit Chung^1, 2, 3^, Zili Gu^1^, Timo Schomann^1, 3^, Xiaoxu Dong^1^, Ruben V. Huis in 't Veld^1, 4^, Marcel G M Camps^5^, Peter ten Dijke^6^, Ferry A. Ossendorp^5^*, and Luis J. Cruz^1^*

**Supplementary Fig. 1** ROS generated by ICG-mediated PDT. (**a**) MC38 cells and (**b**) CT26 colon cancer cells were incubated with ICG (2µg/mL-200µg/mL) for 4 h, followed by PDT. Thereafter, the cells were harvested, washed, and stained with DCFH-DA to determine ROS generation of ICG-PDT. The data show the mean values ± SEM from one representative out of three independent experiments. Statistical significance was calculated using the students *t*-test, by comparing the experimental groups to the control (ns: not significant, p > 0.05, * p < 0.05, ** p < 0.01, *** p < 0.001 and **** p < 0.0001).

**Supplementary Fig. 2** Effects of P407 hydrogel-based PDT when combined with ICIs *in vivo*. (**a**) Individual tumor growth curve of MC38 tumor-bearing mice with associated weight changes of described groups in (**c**); (**b**) Individual tumor growth curve of CT26 tumor-bearing mice with associated weight changes of described groups in (**d**). (ns: not significant, p > 0.05, * or ^#^ p < 0.05, ** p < 0.01, *** p < 0.001 and **** p < 0.0001).

**Supplementary Fig. 3** Analysis of immune cell populations in dLNs and spleen from treated mice. Gating was performed in FlowJo and included only CD45.2^+^ cells. Populations were further gated to include helper T cells (CD3^+^ CD4^+^), CTLs (CD3^+^ CD8^+^), neutrophils (CD11b^+^Ly6G^+^), dendritic cells (CD11b^+^CD11c^+^), and macrophages (CD11b^+^F4/80^+^). (**a**) Helper T cell population in the dLNs; (**b**) CTL population in the dLNs; (**c**) neutrophils, (**d**) dendritic cells, and (**e**) macrophage population in the spleen. Statistical significance was calculated using a one-way ANOVA, by comparing the experimental groups to the control (ns: not significant, p > 0.05, * or ^#^ p < 0.05, ** p < 0.01, *** p < 0.001 and **** p < 0.0001).

**Supplementary Fig. 4** ICG was rapidly absorbed in tumor cells and gets distributed in different cytoplasmic organelles. After cells were incubated with ICG for 4 h, live cells were stained by different organelle markers, then fixed and photographed using fluorescence microscopy. ICG (yellow), Organelles (red) and nuclei (blue), Mito, mitochondria; Lyso, lysosomes; Golgi, Golgi apparatus; ER, endoplasmic reticulum. Scale bar = 50 μm.

**Supplementary Fig. 5** Serum CTLA4 antibody levels at days 0, 1, 3, and 6 following administrations of 50 μg CTLA4-loaded 25% P407 hydrogel.

**Supplementary Fig. 6** Analysis of the therapeutic effect of 25% P407 hydrogel-based CTLA4 therapy. (**a**) Individual tumor growth curves of MC38-tumor-bearing mice during the observed period with associated survival curves in (**c**)**.** (**b**) Individual tumor growth curves of CT26-tumor-bearing mice during the observed period with associated survival curves in (**d**).
